# A case of lupus nephritis in an art student 

**DOI:** 10.5414/CNCS111758

**Published:** 2026-09-07

**Authors:** Kamyar Pournazari, Adrienne Flowers, Sarah H. Khan, Cinthia Drachenberg, Valeriu Cebotaru

**Affiliations:** 1 Division of Nephrology, Departments of Medicine,; 2 Department of Pathology, University of Maryland School of Medicine, and; 3 Division of Nephrology, Departments of Medicine, Baltimore VAMC, Baltimore, MD, USA

**Keywords:** systemic lupus erythematosus, lupus nephritis, acute kidney injury, volatile organic solvents

## Abstract

Systemic lupus erythematosus (SLE) is a complex autoimmune disease that can be triggered by environmental factors. We report a novel case of a 22-year-old female that was exposed to volatile organic solvents (VOCs) while diluting oil-based paint colors prior to presenting with nephrotic syndrome and acute kidney injury, later confirmed as class IV lupus nephritis (LN). Multidisciplinary management resulted in significant clinical improvement, with a return to normal renal function and stabilization of SLE markers. This case highlights possible association between VOCs exposure and LN.

## Introduction 

Systemic lupus erythematosus (SLE) is multisystem autoimmune disease with significant morbidity and mortality that affects 20 – 150 per 100,000 people [1, 2, 3]. SLE is more common in women than in men (9 : 1 ratio) [2, 4, 5] and in people of African descent with prevalence of more than 230 per 100,000 [2]. SLE can affect people of any age with a peak onset between ages of 25 and 45 [1]. Pathogenesis of SLE is multifactorial and complex, and in genetically susceptible people can be triggered by drugs (procainamide and hydralazine having the highest risk) or exposure to toxins such as silica, volatile organic solvents (VOCs), or pesticides [6, 7]. Exposure to VOCs has been associated with membranous and proliferative glomerulonephritis (GN) [8, 9, 10]. Although lupus nephritis (LN) is common in SLE, no direct association between VOCs and LN has been described to best of our knowledge. We report a case of an art student who developed LN after being exposed to VOCs contained in a solvent that was used for dilution of oil-based paint colors. 

## Case presentation 

A 22-year-old female presented with a 10-day history of progressive facial and leg swelling accompanied by dark, frothy urine. Her medical history was remarkable mainly for gingivitis and a recent history of dental problems, managed and treated with amoxicillin for 2 weeks and ibuprofen, 200 mg, 4 times daily for ~ 4 weeks. The patient had no prior past medical history of significance. She denied a history of hypertension, diabetes, or any autoimmune disease. She was an art student who worked with oil-based paints for at least six hours daily, every week, over a period of two years. Her work area was notably a poorly ventilated space with no windows. Oil-based paints were diluted with an agent called Gamsol. This is a highly refined odorless mineral spirit primarily used to thin paints, modify mediums, and clean brushes. Gamsol is a petroleum-based compound that contains heavy hydrotreated naphtha which is a complex combination of hydrocarbons. 

Our patient’s physical exam was significant mainly for bilateral lower extremity edema. The rest of her exam was unremarkable. The initial laboratory findings revealed significantly abnormal renal function tests and immunologic markers. Serum creatinine 1.54 mg/dL, BUN 56 mg/dL, albumin 2.7 g/dL, ANA 1 : 640, dsDNA > 300 IU/mL, C3 < 40 mg/dL, C4 < 8 mg/dL, urine protein 3+, urine RBC > 50/HPF, spot protein-to-creatinine ratio of 20 g/g. ANA, serum creatinine, and urine protein levels were not known at baseline. Hence the patients’ new and acute clinical presentation along with abnormal serology raised suspicion for LN, prompting further investigation. 

On the second day of hospital admission, our patient underwent a renal biopsy. Light microscopy examination revealed a total of 8 glomeruli, with diffuse, global endocapillary and mesangial proliferation and epithelial crescents seen in 2 of the 8 glomeruli. Some of the glomerular loops had an impressive amount of subendothelial immune deposits (Figure 1). Minimal interstitial fibrosis and tubular atrophy was present in 5% of the cortical area. There was no evidence of thrombotic microangiopathy noted in the biopsy. Immunofluorescence demonstrated abundant diffuse and global glomerular deposits of IgG, IgA, IgM, C3, C1q, κ, and λ light chains. Electron microscopy confirmed severe global proliferation in glomeruli and abundant subendothelial, mesangial, and focal epimembranous electron-dense immune complex deposits. Also noted were rare tubuloreticular inclusions along with diffuse effacement of podocyte foot processes. The findings corresponded to class IV LN. EULAR/ACR tool was used for SLE Classification [11], and our patient’s score was 20 (Table 1), indicating severe disease. 

A comprehensive management plan was implemented. She received methylprednisolone 1 g daily for 3 consecutive days, followed by oral prednisone tapering regimen. Mycophenolate mofetil was initiated at 500 mg twice daily and up-titrated to 1,500 mg twice daily. Hydroxychloroquine was introduced but temporarily withheld due to transient elevation of liver enzymes. Supportive measures included bumetanide for volume overload and associated edema, apixaban for anticoagulation to address hypercoagulability linked to nephrotic syndrome, and trimethoprim-sulfamethoxazole for *Pneumocystis jirovecii* pneumonia prophylaxis, with subsequent switch to dapsone due to adverse reactions. Ibuprofen was discontinued. 

The patient demonstrated marked clinical and biochemical improvement over 6 months. Renal function normalized, with serum creatinine stabilizing at 0.6 mg/dL, proteinuria resolved with a spot protein-to-creatinine ratio of 0.1 g/g, serum albumin levels normalized to 4.0 g/dL, complements C3 and C4 levels returned to normal ranges, while dsDNA antibodies remained positive. The patient transitioned to maintenance therapy with mycophenolate mofetil 1,000 mg twice daily, hydroxychloroquine 300 mg daily, and belimumab once a week achieving sustained remission. The patient was followed for 2.5 years until she relocated. 

## Discussion 

SLE is a multisystem autoimmune disease with an incompletely understood pathogenesis. Approximately half of the patients with SLE will develop LN. Although exposure to VOCs have been associated with SLE [7, 12], no association between VOCs and LN have been described to date. Previous case reports described an association between VOCs and GN such as proliferative, anti-glomerular basement membrane (anti-GBM), and membranous. Beirne and Brennan [9], described 5 cases of anti-GBM associated with VOCs exposure. Zimmerman et al. [8], described 4 cases of anti-GBM and 8 cases of proliferative GN associated with VOCs exposure. Four out of 7 patients with proliferative GN had glomerular deposits of IgG, IgM, and C3 [8] (1 patient did not have immunostaining). Ehrenreich et al. [10], described 4 cases of membranous GN associated with VOCs exposure. We report a case of LN in an art student who was working with oil-based paint colors that were diluted with Gamsol which contains VOCs. 

This patient was taking ibuprofen, which is an nonsteroidal anti-inflammatory drug (NSAID). NSAIDs have been linked to a spectrum of renal pathologies such as acute kidney injury (hemodynamically mediated or acute tubular necrosis), acute papillary necrosis, acute interstitial nephritis, hypertension and edema, minimal change disease, membranous GN, focal sclerosis, focal and proliferative GN, and anti-GBM nephritis [13, 14]. To the best of our knowledge, there is no described association between NSAIDs and LN and therefore it is less likely that ibuprofen alone would trigger LN. It is possible that by decreasing the glomerular filtration rate, ibuprofen increased the blood level of VOCs which could have contributed to LN in this patient. 

This patient had gingivitis that was treated with amoxicillin. While viral pathogens such as Epstein-Barr virus have been associated with the development of SLE [15], there is limited evidence to suggest that bacterial infections can trigger SLE [16]. 

Oil paints contain heavy metals such as cadmium, cobalt, and chromium that can be nephrotoxic. These heavy metals cannot be inhaled from oil paints, but can be inhaled during sanding, but this was not the case for our patient. Therefore, cadmium, cobalt, and chromium are less likely to be implicated in this patient’s renal disease. Chronic inhalation of VOCs activates reactive oxygen species that will activate a complex inflammatory pathway that will lead to autoimmune diseases [6]. Two studies examined high-dose exposure to VOCs and found no dose–response association with SLE [12, 17] indicating that even low-dose exposure can be associated with SLE. Based on the safety data sheet, Gamsol may be fatal if it enters the airways and needs to be stored in well-ventilated places. Gamsol contains low amounts of VOCs that can accumulate in closed spaces. Our patient was working in a poorly ventilated space without windows and likely inhaled high volumes of VOCs from Gamsol which could theoretically trigger SLE. 

The pathogenesis of SLE/LN is poorly understood, and it is thought to be multifactorial. There are at least two possible mechanisms that would link VOCs to SLE/LE. Circulating levels of TNF-α are elevated during active disease [18]. In vitro studies showed that Toluene, an aromatic hydrocarbon, increased TNF-α production in human peripheral blood mononuclear cells suggesting a possible mechanism for SLE/LN [19]. Exposure to hydrocarbons can activate cytokines such as CD70, CD154, IL-4, IL-6 and CD9, MMP9 via DNA hypomethylation that could lead to SLE [6], suggesting another possible mechanism. There are likely other mechanisms linking VOCs to SLE/LN and this needs to be explored in future studies. 

It is unlikely that NSAID use and dental infection are associated with LN along with VOCs exposure, but this cannot be completely ruled out. 

This case indicates a possible association between VOCs exposure and LN and complemented previous reports that showed an association between VOCs exposure and SLE. In addition, it highlights the possible risk of LN in occupational exposure to VOCs in professional artists that work with oil-based paint colors. 

## Data availability 

All data generated or analyzed during this study are included in this article. Further enquiries can be directed to the corresponding author. 

## Authors’ contributions 

Research idea and study design: KP, AF, VC; data acquisition: KP, AF, SK, CD, VC; data analysis/interpretation: KP, AF, SK, CD, VC; supervision or mentorship: VC. VC takes responsibility that this study has been reported honestly, accurately, and transparently, and accepts accountability for the overall work by ensuring that questions pertaining to the accuracy or integrity of any portion of the work are appropriately investigated and resolved. 

## Funding 

No funding was received. 

## Conflict of interest 

The authors have no conflict of interest to declare. 

**Figure 1:**
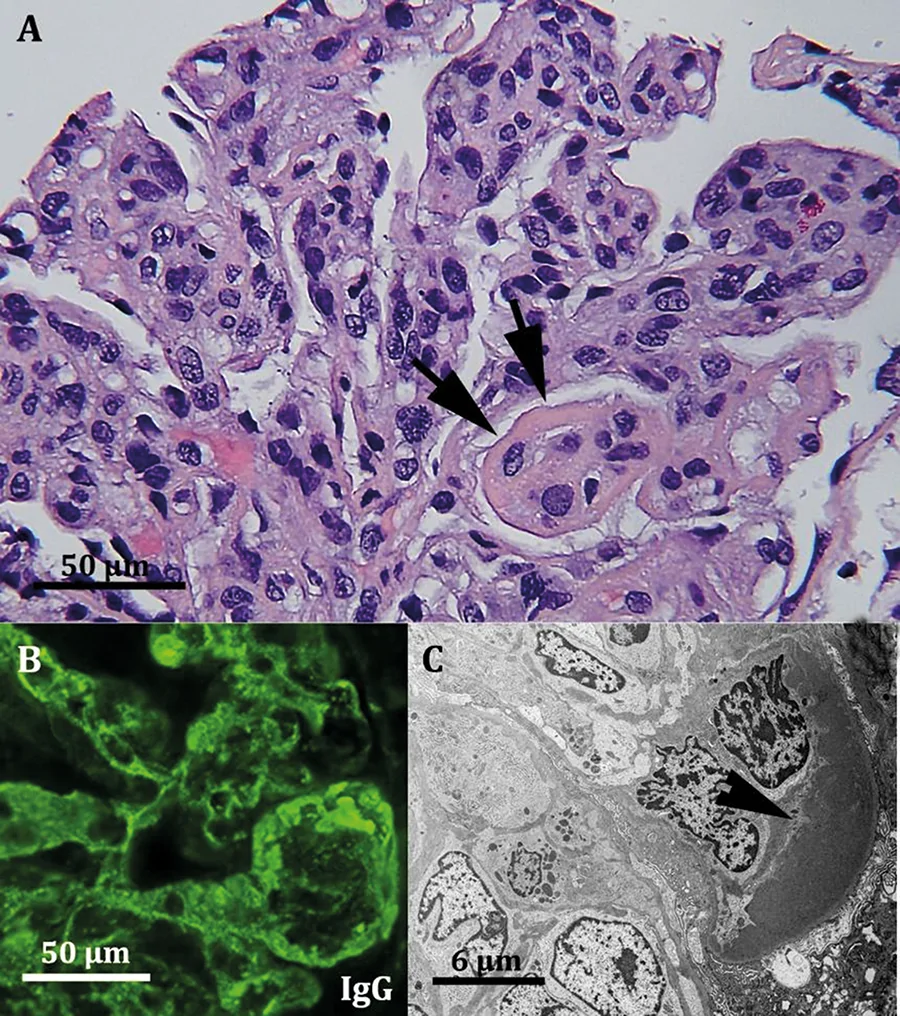
Renal biopsy. A: H&E stain shows global endocapillary proliferation with accentuation of glomerular lobularity. Massive subendothelial immune deposition leads to markedly thickened capillary walls (arrows). B: Immunofluorescence stain for IgG shows diffuse staining within mesangium and outlining the capillary walls. All other stains showed the same pattern of deposition (full-house staining pattern). C: Electron micrograph illustrates severe endocapillary proliferation leading to loss of patent capillary lumina. On the right, a large subendothelial immune deposit outlines the capillary wall (arrowhead).

**Table 1.** EULAR/ACR classification criteria for systemic lupus erythematosus.

**Clinical domains and criteria**	**Weight**
Renal biopsy class III or IV lupus nephritis	10
Low C3 and low C4	4
Anti-dsDNA antibody	6
Total score	20
